# Report of the fifth meeting of the European Consortium 'Care for CMMRD' (C4CMMRD), Leiden, The Netherlands, July 6th 2019

**DOI:** 10.1007/s10689-020-00194-1

**Published:** 2020-07-02

**Authors:** M. Suerink, K. Wimmer, L. Brugieres, C. Colas, R. Gallon, T. Ripperger, P. R. Benusiglio, E. M. A. Bleiker, Z. Ghorbanoghli, Y. Goldberg, J. C. H. Hardwick, M. Kloor, M. le Mentec, M. Muleris, M. Pineda, C. Ruiz-Ponte, H. F. A. Vasen

**Affiliations:** 1 Department of Clinical Genetics, Leiden University Medical Centre, Leiden, The Netherlands; 2 Division of Human Genetics, Medical University of Innsbruck, Innsbruck, Austria; 3 Child and Adolescent Cancer Department, Gustave Roussy Cancer Campus, Villejuif, France; 4 Department of Genetics, Institut Curie, Université de Recherche Paris Sciences et Lettres, Paris, France; 5 Faculty of Medical Sciences, Newcastle University, Newcastle upon Tyne, UK; 6 Department of Human Genetics, Hannover Medical School, Hannover, Germany; 7 Sorbonne Université, Inserm, Unité Mixte de Recherche Scientifique 938, Equipe Instabilité Des Microsatellites et Cancer Centre de Recherche Saint-Antoine, CRSA, Paris, France; 8 Unité Fonctionnelle d’Oncogénétique, Département de Génétique et Institut Universitaire de Cancérologie, Groupe Hospitalier Pitié-Salpêtrière, AP-HP, Sorbonne Université, 75013 Paris, France; 9 Division of Psychosocial Research and Epidemiology & Family Cancer Clinic, The Netherlands Cancer Institute, Amsterdam, The Netherlands; 10 The Netherlands Foundation for the Detection of Hereditary Tumours, Leiden, The Netherlands; 11 Department of Gastroenterology and Hepatology, Leiden University Medical Centre, Leiden, The Netherlands; 12 Raphael Recanati Genetics Institute, Rabin Medical Center, Beilinson Campus, Petah Tikva, Israel; 13 Department of Applied Tumor Biology, Institute of Pathology, University Hospital Heidelberg, Heidelberg, Germany; 14 Clinical Cooperation Unit Applied Tumor Biology, DKFZ (German Cancer Research Center) Heidelberg, Heidelberg, Germany; 15 Hereditary Cancer Program, Catalan Institute of Oncology, ONCOBELL Program, Institut d’Investigació Biomèdica de Bellvitge (IDIBELL), Hospitalet de Llobregat, Barcelona, Catalonia Spain; 16 Centro de Investigación Biomédica en Red de Cáncer (CIBERONC), Madrid, Spain; 17 Fundacion Publica Galega de Medicina Xenomica, SERGAS, Instituto de Investigacion Sanitaria de Santiago (IDIS), Grupo de Medicina Xenomica-USC, Centro de Investigacion Biomedica en Red de Enfermedades Raras (CIBERER), 15706 Santiago de Compostela, Spain

Constitutional mismatch repair deficiency (CMMRD) is an autosomal recessive condition associated with a high risk of cancer in children, adolescents and young adults. CMMRD is caused by homozygous or compound heterozygous pathogenic germline variants in one of four mismatch repair (MMR) genes (i.e., *MLH1, MSH2, MSH6 *and *PMS2*) [1], whereas mono-allelic (heterozygous) MMR gene variants result in autosomal dominant Lynch syndrome [2].

Lynch syndrome is one of the most common cancer predisposition syndromes and in adults leads to an increased risk of colorectal cancer, endometrial cancer and other malignancies [2]. By contrast, CMMRD is rare and leads to an increased risk of brain tumors, hematological malignancies, colorectal cancer and a wide range of other cancers in children, adolescents and young adults [1]. In addition, most patients with CMMRD have non-neoplastic features, with multiple café-au-lait maculae (CALM) being the most prevalent [1, 3].

This report summarizes the 5th meeting held by the ‘Care for CMMRD’ (C4CMMRD) consortium in Leiden, the Netherlands, on July 6th 2019. The consortium was established in 2013 with a number of explicit goals, including improving the care of CMMRD patients and their families, increasing knowledge and awareness of the syndrome, developing guidelines for diagnosis and clinical care, establishing a database to record clinical details of known patients with CMMRD, and conducting collaborative studies. Meetings are held every 1 to 2 years with the aim of updating members on the latest results and developments from ongoing research, and initiating new study proposals. Thirty-five participants from nine countries and various medical fields (including basic and translational researchers, pediatric oncologists, clinical geneticists, gastroenterologists, a psychologist and molecular geneticists) attended the meeting.

## CMMRD database in Paris

For research purposes, a CMMRD patient database was established at the Gustave Roussy Cancer Campus in Villejuif, France. To kick off the meeting, Chrystelle Colas gave an update on its current status*.* At the time of the meeting, 87 CMMRD patients from 66 families had been included, of whom 27 were still alive (age range 3–48 years). The largest number of pathogenic variants were identified in *PMS2* (n = 34), followed by *MSH6* (n = 19), *MSH2* (n = 8) and *MLH1* (n = 4). Molecular results were lacking for one patient. All but one patient developed at least one malignancy. Of 154 tumor diagnoses in 86 patients, tumors of the central nervous system were the most frequent (n = 64, 42%), followed by hematological malignancies (n = 45, 29%; mainly T lymphoblastic lymphomas), and Lynch syndrome-related malignancies (n = 43, 28%).

## Role of functional assays

The previously developed C4CMMRD criteria defining clinical suspicion of a CMMRD diagnosis in young cancer patients [1] were designed to have high diagnostic sensitivity at the cost of specificity. Detection of pathogenic variants in both alleles of an MMR gene is required to confirm the diagnosis. A definitive molecular diagnosis or, equally important, a rejection of this diagnosis is also needed when testing for CMMRD, when it is the differential diagnosis to neurofibromatosis type 1 (NF1)/Legius syndrome in a malignancy-free child with NF1 signs without a causative *NF1* or *SPRED1* pathogenic variant [3]. Unfortunately, molecular genetic testing is not always conclusive, and the diagnosis of CMMRD is frequently confounded by MMR variants of unknown significance (VUS) and *PMS2* pseudogenes.

The need to resolve diagnostic ambiguities has led to the development of functional CMMRD assays and highly sensitive microsatellite instability (MSI) assays that detect low‐frequency microsatellite length variants in non-neoplastic tissues, a diagnostic hallmark of CMMRD.

Current functional approaches include assessing methylation tolerance in combination with MSI in primary lymphoblastoid cell lines [4] and assessment of the MMR functionality of protein extracts from patient cells [5]. Martine Muleris presented data on the methylation tolerance test as performed in 85 patients with a CMMRD-like phenotype and 92 controls. It has been previously shown in a smaller cohort that this test can discriminate CMMRD patients and healthy controls and may therefore be a useful diagnostic tool in CMMRD-like patients [4]. The results of the methylation tolerance test in this larger cohort will be published elsewhere.

Although reliable, functional assays performed in specialized laboratories may not be easily scalable. Another drawback is that they require fresh patient material [4, 5]. MSI assays for CMMRD detection can also be applied to patient DNA in retrospective studies and likely require less specialized laboratories. The first MSI assay, which assesses low-level MSI in three dinucleotide repeat markers in patient peripheral blood leukocytes (PBLs), is simple, fast and scalable, but has the disadvantage of being insensitive to MSH6 deficiency due to the type of microsatellite analyzed [6].

At the meeting, Richard Gallon presented a sensitive and scalable MSI assay that detects low-level MSI in patient PBLs using 24 mononucleotide repeat markers. The assay method was developed in a pilot cohort of 5 CMMRD patients and 40 controls, and was validated by analyzing an additional 27 CMMRD patients and 54 controls, in a blinded manner, as well as 40 Lynch syndrome patients. The assay achieved 97% sensitivity and 100% specificity, including the detection of MSH6-deficient patients and patients with hypomorphic *PMS2* variants [7]. The single false negative result was attributed to the patient’s chemotherapy-induced aplasia when this sample was collected, as additional samples collected from the same patient after recovery from aplasia were correctly classified [7].

Marta Pineda presented a high-sensitivity MSI (hs-MSI) assay that can be used in non-neoplastic tissues of Lynch syndrome and CMMRD carriers and is based on a panel of 186 mononucleotide repeat markers. This approach was applied to a training cohort including 15 blood samples from negative controls, 48 from Lynch syndrome individuals and 12 from CMMRD patients. The MSI score was significantly higher in blood DNA samples from CMMRD patients compared to healthy controls, and without any overlap. This finding was confirmed using a validation set including 36 blinded samples (18 controls and 18 CMMRD provided by the C4CMMRD consortium) and reached 100% specificity and sensitivity, even in the case of MSH6-deficient patients. Moreover, blood from germline *TP53, POLE, POLD1* and *NF1* pathogenic variant carriers and early-onset Lynch syndrome cases did not show high hs-MSI scores, demonstrating that the assay discriminates between CMMRD and other hereditary syndromes with overlapping phenotypes. The results of this approach also showed good correlations with the MSI assay presented by Richard Gallon. This work has been recently published by González-Acosta et al*. *[8].

Patrick Benusiglio presented a proof-of-concept study of another assay detecting ultra-low MSI in leukocytes, thus enabling rapid and accurate diagnosis of CMMRD. This study will be published elsewhere.

In conclusion, several reliable MSI assays aimed at rapid diagnosis of CMMRD have been developed with the support of the C4CMMRD consortium and at least one is now suitable for scalable screening of at‐risk populations (see proposal for the assessment of *Prevalence of CMMRD in patients with T-cell acute lymphoblastic lymphoma)*.

## CMMRD-like phenotypes

Differential diagnoses in patients with a “CMMRD-like” phenotype, in whom neither identification of biallelic germline MMR pathogenic variants nor functional or MSI assays could confirm the diagnosis, were another topic of the 5th C4CMMRD meeting.

Clara Ruiz-Ponte presented the case of a boy who fulfilled the C4CMMRD criteria for a suspected diagnosis of CMMRD. This boy, with a maternal family history of Lynch syndrome, developed colorectal cancer at 12 years of age and had a skin nodule suspected to be a neurofibroma. However, he only carried a maternally-inherited pathogenic *MSH2* variant and was negative for CMMRD based on all functional and MSI assays used. Therefore, other possible scenarios were explored that could explain the early age of tumor onset. Interestingly, a number of paternally-inherited low/moderate penetrance variants in other cancer predisposing genes and genes described as genetic modifiers of Lynch syndrome were identified. The assembled data on this patient suggest that the combination of several low-risk modifier alleles together with a pathogenic *MSH2* variant may be responsible for the CMMRD-like phenotype in this patient [9].

Katharina Wimmer presented three cases with a “CMMRD-like” phenotype likely explained by germline *POLE* pathogenic variants. These included a previously published case of a 14-year-old boy with colorectal cancer, colon adenomas, a pilomatricoma and multiple CALM [10], and two unpublished cases, one a 31-year-old male with colorectal cancer, adenomatous polyposis, glioblastoma, CALM and pilomatricomas, and the other a 4-year-old girl with a malignant central nervous system tumor and CALM. As was the case for a *POLE* pathogenic variant found in a medulloblastoma patient with a “CMMRD-like” phenotype which was published after our meeting [11], the *POLE* variants found in all three patients presented at the meeting were de novo and were previously seen as somatic but never as germline mutations. Taken together, these cases support the evolving notion that specific *POLE* exonuclease domain variants, typically seen as somatic variants in hypermutated tumors, confer a phenotype reminiscent of CMMRD resulting from a germline pathogenic variant.

Katharina Wimmer also presented two siblings, diagnosed with bowel cancer as teenagers, who both had a maternally-inherited, heterozygous *PMS2* pathogenic variant and a paternally-inherited *POLD1* variant likely to affect POLδ exonuclease activity. This suggests that the “CMMRD-like” phenotype can be caused by digenic inheritance of MMR and polymerase proofreading inactivating mutations.

Marine Le Mentec and Chrystelle Colas presented a patient with duodenal cancer at age 17 with a maternally-inherited heterozygous *PMS2* pathogenic variant, as well as a paternally-inherited heterozygous *POLE* variant of unknown significance.

Taken together, these cases demonstrate that sequencing of *POLE* and *POLD1* should be considered in patients with a “CMMRD-like” phenotype in whom CMMRD cannot be confirmed (either molecularly or functionally).

## CMMRD and early-onset systemic lupus erythematosus

As listed in the C4CMMRD consensus guidelines, there are a number of (non-)neoplastic features, such as pigmentation alterations, pilomatricomas and vascular anomalies, that are indicative of CMMRD in the (young) cancer patient or in a patient with suspected NF1 but without an *NF1* or *SPRED1* pathogenic variant [1, 3]. At the meeting, Yael Goldberg introduced a new non-neoplastic feature by presenting two cases of young children with CMMRD and pediatric systemic lupus erythematosus (SLE). Age of onset was 5 years in both children, and one child did not have any cancer at the time of diagnosis [12]. Together with three previously described children with CMMRD and SLE [13–15], these cases indicate that pediatric onset SLE should be considered a diagnostic criterion of CMMRD, and CMMRD testing should be offered if additional features are present [1]. This might aid early diagnosis, but treatment of SLE in these patients may be challenging as the immune checkpoint inhibitors currently under investigation as a treatment for CMMRD-related cancers could cause SLE to flare, while steroid treatment for SLE may lessen the effect of immune checkpoint inhibitors.

## Psychological impact

Eveline Bleiker was invited to the meeting to present and discuss her experience of the psychological impact of another severe cancer predisposition syndrome, Li-Fraumeni syndrome (LFS), and reflect on possible lessons for CMMRD. Based on experience and the LFS literature, a high uptake of genetic testing is expected in those who are aware of a possible hereditary risk, particularly the siblings of affected children, and it is probable that 20–30% of patients with a confirmed diagnosis will experience high levels of distress. However, the large majority of all patients likely experiences specific worries related to CMMRD and to coping with cancer in their family. Professional psychosocial support should be offered to all. Worries regarding cancer risk in children are also likely to be high and deserve the attention of a counselor and, if needed and preferred, a professional psychosocial worker. The Psychological Aspects of Hereditary Cancer questionnaire can be used as a tool to identify and discuss any specific problems experienced [16]. All of these expectations are based on studies of LFS. To learn more about the psychosocial issues that accompany CMMRD, qualitative and quantitative studies on this topic in this population are recommended.

## Experience with colonoscopic surveillance

James Hardwick outlined his experience of performing colonoscopic surveillance in a CMMRD patient in Leiden. Surveillance commenced at 26 years of age and was performed yearly for 4 years, until the patient developed a glioblastoma. At the first colonoscopy a 2 cm villous adenoma with high grade dysplasia was successfully removed by piecemeal Endoscopic Mucosal Resection. A 1 cm sessile serrated polyp with low grade dysplasia and 2 sub-centimeter adenomas were also removed. Subsequent colonoscopies were performed using chromoendoscopy due to the subtle, flat morphology of several of the polyps, which led to the removal of several more sub-centimeter sessile serrated polyps and adenomas over a 4-year period. It can be concluded that the colon is at high risk in CMMRD. Serrated polyps and classical adenomas are both found, and advanced polyps can be removed successfully endoscopically, so intensive surveillance seems justified.

## Proposals for collaborative studies and some recent results

### Selection criteria for CMMRD testing in children without malignancy with an NF1-like phenotype

CMMRD is a valid differential diagnosis in children without cancer who are suspected of sporadic NF1 but in whom no causative *NF1* or *SPRED1* variant has been identified. In 2019, a consensus guideline was published by the C4CMMRD consortium that advocated testing of CMMRD in preselected patients with a higher a priori risk, rather than reflex testing of all suspected sporadic NF1 children lacking causative *NF1*/*SPRED1* variants [3]. Manon Suerink and Katharina Wimmer presented the design of a prospective multicenter study to validate the specificity of the criteria by prospectively documenting cases to whom CMMRD testing is offered.

### PD-1 blockade as a treatment in CMMRD

Laurence Brugieres gave a presentation on the potential of PD-1 blockade as a treatment for CMMRD-related cancer. MMR-deficient cancers have been shown to respond well to this treatment modality [17]. To evaluate the proportion of patients that might benefit from treatment with PD-1 inhibitors and to analyze indications and efficacy of immunotherapy in this first set of patients, an analysis of the patients included in the C4CMMRD database was undertaken. In addition, collaborating researchers were contacted and asked to include additional patients who received immunotherapy.

In total, 18 CMMRD patients treated with PD-1 inhibitors were identified, and high-grade glioma was the indication for immunotherapy in 13 patients (2 for front-line treatment, 11 at relapse). Type of treatment was known for 17 of the 18 patients: pembrolizumab for 5 and nivolumab for 12 patients (3 of which are included in a trial combining ipililumab and nivolumab). Ten patients had progressive disease, while 8 patients showed stabilization and/or a response. Following initiation of immunotherapy, 11 patients died after a median survival of 5 months (9 high-grade gliomas and 2 digestive tract cancers) and 7 patients were still alive, with a median follow-up of 20 months (4 high-grade gliomas, 2 digestive tract cancers and one non-Hodgkin lymphoma).

From this short series of patients it appears that, despite a high mutation burden, not all CMMRD patients benefit from immunotherapy. It was proposed to include this series of patients in the SIGN’it project, an on-going project aimed at identifying biomarkers associated with the response to PD-1 inhibitors (B. Geoerger, France). To collect more data, a specific data sheet will be sent to all investigators who have included a CMMRD patient treated with immunotherapy in the C4CMMRD database.

### Guidelines for genetic counseling

Tim Ripperger followed with a presentation drawing attention to the need for guidelines regarding counseling issues faced by genetic counselors, clinical geneticists, and oncologists involved in the care of CMMRD families. Following on from the consortiums’ focus on the development of surveillance guidelines [18] and clinical criteria indicating when to test for CMMRD in cancer patients [1], as well as the recent refinements concerning individuals with suspected neurofibromatosis type 1 but without an identifiable *NF1* or *SPRED1* pathogenic variant [3], we discussed and agreed on the need for genetic counseling recommendations for families with suspected and/or diagnosed CMMRD. Although there is a growing body of literature dealing with CMMRD, none of the papers specifically address counseling issues (e.g., ethical and legal issues related to predictive testing in minor siblings, or the follow-up of parents with a formal molecular diagnosis of Lynch syndrome in the absence of a family history of Lynch syndrome-associated malignancies). Moreover, we need to address the question of whether CMMRD should be integrated into the counseling of Lynch syndrome patients, and if so, when and how this integration should take place.

### Vaccination

Matthias Kloor gave an update on the role of vaccination in the prevention of cancer in Lynch syndrome and posed questions that need to be answered regarding a similar vaccination for CMMRD: (1) What are the neoantigen profiles of CMMRD-associated tumors? (2) Is there a pre-existing systemic immune response in CMMRD? (3) What immune response pathways are active in CMMRD, and can auto-immune symptoms be expected? (4) What are the mechanisms of immune evasion in CMMRD tumors?

### Prevalence of CMMRD in patients with T-cell acute lymphoblastic lymphoma

Richard Gallon proposed the use of a newly developed, scalable MSI assay (see above) to study the prevalence of CMMRD in children with T-cell acute lymphoblastic lymphoma (T-LBL) and high-grade gliomas using PBLs from retrospective cohorts of patients with these types of cancer.

### Preliminary results of surveillance according to the C4CMMRD guidelines

Zeinab Ghorbanoghli presented the preliminary results of surveillance according to the protocol as proposed by the C4CMMRD consortium in 2014 [18]. Data were collected from 22 patients, including 12 females. Fifteen of these patients (68%) had biallelic *PMS2* variants. Seventy-seven percent of the patients had developed a previous cancer, including mainly colorectal and hematological tumors. Over a follow-up period of up to 5 years, 15 malignancies developed amongst 12 patients. These malignancies were most frequently located in the digestive tract, followed by brain tumors. Twelve patients were diagnosed with (multiple) adenomas in the colon. The investigators concluded that the yield of screening was very high. The preliminary results suggest that surveillance of the digestive tract is effective because it leads to the endoscopic removal of many polyps and the detection of early cancers. However, the benefit of screening of the brain remains uncertain.

Following this presentation, a discussion arose regarding whether surveillance guidelines should be modified in view of these findings. One participant suggested future recommendation of brain MRI screening at intervals of 6 months rather than the 6–12 months currently advised. In addition, the question came up of whether an earlier starting age for colonic surveillance (currently 8 years) should be considered given that adenomatous polyps have been reported in patients below this age. After the final analysis of the data, possible changes to the protocol will be discussed again.
